# The recent genetic modification techniques for improve soil conservation, nutrient uptake and utilization

**DOI:** 10.1080/21645698.2024.2377408

**Published:** 2024-07-15

**Authors:** Gideon Sadikiel Mmbando, Kelvin Ngongolo

**Affiliations:** Department of Biology, College of Natural and Mathematical Sciences, The University of Dodoma, Dodoma, United Republic of Tanzania

**Keywords:** Genetic interventions, nutrient transporters, root architecture, soil erosion, soil fertility, sustainable agriculture

## Abstract

Advances in genetic modification (GM) techniques have generated huge interest in improving nutrient utilization, maximizing nutrient uptake, and conserving soil in the pursuit of sustainable agriculture. Unfortunately, little is still known about the recent advancements in the application of GM tactics to enhance each of these areas. This review explores the latest GM strategies intended to support soil conservation, maximize nutrient uptake, and improve nutrient utilization in farming, highlighting the critical roles that soil health and nutrient management play in sustainable farming. GM strategies such as improving the efficiency of nutrient uptake through enhanced root systems and increased nutrient transport mechanisms are well discussed. This study suggests that addressing potential obstacles, such as ethical and regulatory concerns, is a necessity for long-term sustainability applications of GM technologies to raise agricultural yields.

## 1. Introduction

Agriculture is at a critical crossroads due to growing environmental pressures and the need to feed a growing global population.^1,2^ Our ability to minimize soil degradation, maximize nutrient utilization efficiency, and optimize nutrient uptake will determine how sustainable our food production systems will be.^3–5^ Within this framework, genetic modification (GM) methods have become effective instruments for tackling these pressing issues.^6^ Furthermore, while soil conservation is essential to sustainable agriculture, the widespread erosion and degradation of arable land represent a serious threat to the world’s ability to produce food.^7^ Although somewhat successful, traditional soil conservation techniques frequently fail to keep up with the increasing severity of climate extremes and unsustainable land management techniques.^8,9^

Genetic modification, or genetic engineering, is a process that allows foreign DNA to be added to a host plant’s genetic makeup.^10,11^ With its ability to increase soil structure, decrease erosion, and increase agricultural ecosystem resilience, GM presents a viable way to improve soil conservation efforts.^6,12^ Moreover, a crucial component of crop productivity and nutritional quality is plant nutrient uptake.^13^ However, ineffective soil nutrient acquisition reduces crop yields and, by using too much fertilizer, contributes to pollution in the environment.^5,14^ GM tactics that target nutrient uptake mechanisms, like root architecture enhancement and improved nutrient transport processes, have great potential to boost nutrient acquisition efficiency while lowering agriculture’s environmental impact.^6,12,15^ Moreover, genetic, physiological, and environmental factors all play a role in the intricate process of how plants use nutrients.^16^ Crop yields are jeopardized by inefficient nutrient utilization, which also exacerbates soil degradation, greenhouse gas emissions, and nutrient runoff.

Although GM techniques for soil conservation, nutrient uptake, and utilization are gaining popularity and advancing, there is still a lack of thorough reviews that combine these three important aspects. The literature that is currently available frequently concentrates on specific elements, like nutrient uptake or soil conservation, without offering a comprehensive picture of how GM techniques can address several issues at once.^6,17,18^ Further research is also necessary to examine the possible trade-offs and synergies between various genetic interventions in soil-plant systems. Closing this gap would guide future research efforts in this area and advance our understanding of the role of GM in sustainable agriculture.

Conventional farming methods do not effectively address soil erosion, ineffective nutrient uptake, and inadequate nutrient utilization, which ultimately results in lower crop yields and greater environmental damage. For environmentally friendly food production and conservation, these issues must be resolved.^8,9,19^ New developments in GM techniques have the potential to improve soil conservation, optimize nutrient uptake, and increase the efficiency of nutrient utilization. To create sustainable farming methods that guarantee food security while reducing environmental impact, it is crucial to comprehend the potential of these approaches. On the other hand, little recent data exists on the application of GM to enhance nutrient uptake, utilization, and soil conservation.

In this study, 134 journal articles were selected at random from PubMed, ScienceDirect, and Google Scholar papers. This review looks at the efficacy of different genetic interventions to find strategies that show promise for optimizing plant nutrient acquisition, boosting soil health, and increasing nutrient use efficiency. This study sheds light on how GM technologies can support environmentally friendly farming practices and lessen the harm caused by the erosion and degradation of arable land.

## 2. GM Approaches for Enhancing Soil Conservation and Nutrient Uptake

**Figure 1.**
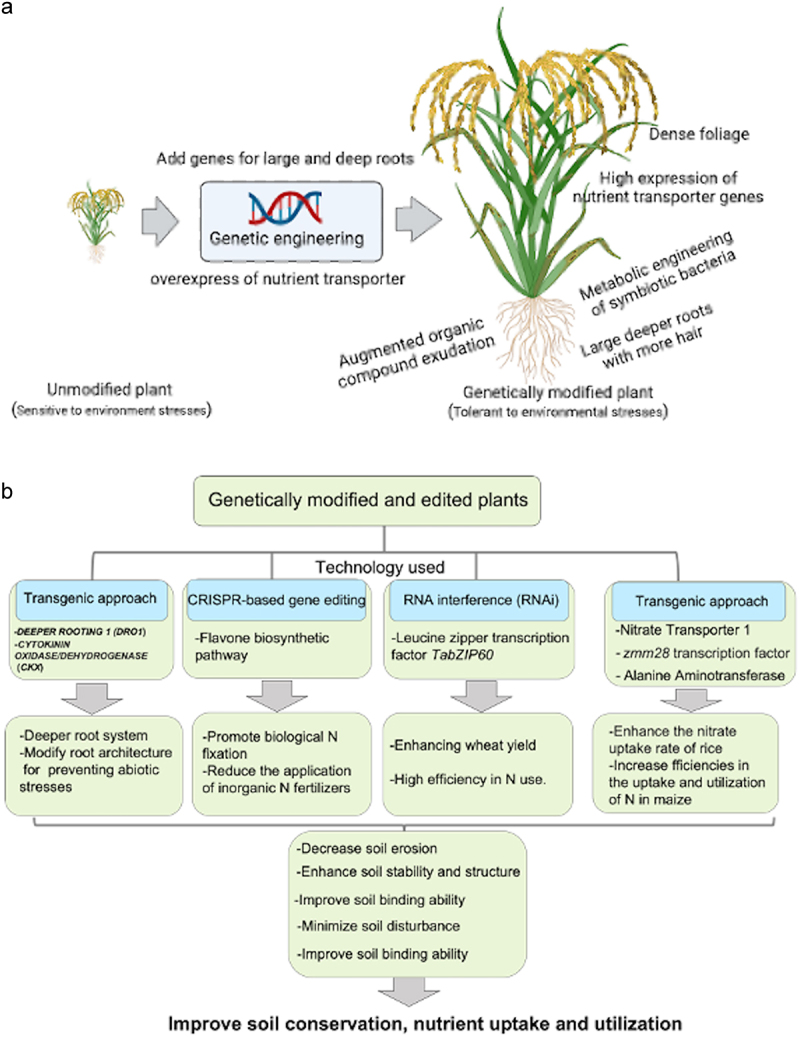
The benefit of genetically modification technologies on soil conservation and nutrient uptake and utilization. (a) Genetically modified (GeMo) plants for improving soil conservation are produced by the process of genetic engineering through the addition of genes for large and deeper root systems or overexpression of nutrient transporter genes. (b) The GeMo produced by transgenic approach and RNA interference (RNAi) or edited plant produced by gene editing technique such as clustered regularly interspaced short palindromic repeats (CRISPR)-associated endonuclease Cas9 (CRISPR-Cas9) with a large, deeper root, high biological N fixation, high efficiency in use N, and enhancing nitrate uptake will improve nutrient absorption and soil binding ability. Its ability to tolerate environmental pressure such as abiotic stresses will enable the plant to survive harsh conditions and thereby protect the soil from degradation. Genetic modification of plants to produce organic compound exudation from roots will enhance soil stability and structure. All of these genetic modification pathways improve soil conservation, nutrient uptake, and utilization, which eventually improve agriculture yield and ecological sustainability.

Sustainable land management techniques that aim to maintain and safeguard the integrity of soil resources must include soil conservation as a fundamental element. The soil is an essential natural resource that keeps life on Earth alive by controlling water flow, supplying nutrients for plant growth, and fostering biodiversity.^20,21^ However, agricultural productivity, ecosystem health, and global supply of food are seriously threatened by soil erosion, degradation, and loss.^22,23^ A variety of tactics and methods are included in soil conservation, with the goals of halting soil erosion, enhancing soil structure, and preserving soil fertility (Figure 1).^24,25^ These tactics involve minimizing soil loss from wind and water erosion by using erosion control techniques like terracing, contour plowing, and the use of vegetative cover. To further reduce soil disturbance and preserve soil organic matter, conservation tillage techniques, reduced tillage intensity, and promotion of sustainable land use practices are all part of soil conservation practices.^26,27^ Because GM techniques increase the resilience and adaptability of endangered plant species, they are vital to plant conservation efforts. For example, genetically modified (GeMo) plants are able to flourish in a variety of challenging environments by introducing genes that provide resistance to pests, diseases, and environmental stressors.^17,28–30^ The preservation of genetic diversity within species is made possible by this technology, and this is essential for the durability and stability of ecosystems. Furthermore, by reintroducing features that enhance survival and reproduction, GM can be used to replenish plant populations that have declined as a result of habitat loss or climate change. Furthermore, it permits the development of plants with enhanced characteristics that can fit into various ecological niches, protecting ecosystem sustainability. GM produces plants with characteristics that preserve and enhance soil health, greatly enhancing soil conservation practices. For example, GeMo plants can be modified to have stronger, deeper root systems, which better anchor the soil and reduce soil erosion. Therefore, GM is a potent tool that supports conventional soil conservation techniques by providing creative ways to protect plant biodiversity and uphold ecological balance. As a result, soil conservation is essential for encouraging sustainable farming, preserving natural areas, and lessening the effects of climate change.^31^ We can guarantee the agricultural systems’ long-term sustainability and protect soil resources for upcoming generations by implementing soil conservation practices.

In plants, the uptake of nutrients is a basic process that is necessary for growth, development, and general physiological health. Through their root systems, plants absorb vital nutrients from the soil solution, such as micronutrients, phosphorus (P), potassium (K), and nitrogen (N).^32,33^ Nutrient ions are absorbed by the roots, transported across root cell membranes, and then translocated to different plant tissues through the xylem and phloem.^34^ Plant-specific characteristics like root morphology, physiology, and genetic traits, along with environmental factors like pH, moisture content, and soil nutrient availability, all work together to control nutrient uptake.^35–37^ To maximize crop yields, nutritional quality, and general plant health, efficient nutrient uptake is essential.

Genetic techniques provide novel ways to support conservation efforts in soil by focusing on important plant characteristics that affect soil stability and erosion resistance.^6,12,38^ Modifying root architecture is one genetic strategy to improve soil-binding ability and decrease soil erosion.^15,39,40^ The modified plants will have the ability to anchor soil particles and stop wind and water erosion by encouraging the growth of larger and deeper root systems (Figure 1(a)).^41–43^ An additional genetic strategy aims to improve ground cover and decrease soil exposure to erosive forces by optimizing biomass allocation and plant canopy structure (Figure 1(b)).^44–47^ Plants have been modified to produce a canopy layer that protects the soil surface from wind and rainfall, reducing erosion, by undergoing genetic modifications that encourage denser foliage and increased biomass production. In addition, genetic interventions that target the improvement of plant resistance to environmental stressors like drought, strong winds, ultraviolet-B (UV-B) radiation, and so on^10,48–50^ can indirectly support soil conservation by preserving vegetation cover and minimizing soil disturbance during extreme weather events (Figure 1). Thus, genetic methods have the potential to improve soil conservation by reducing the risk of erosion and encouraging the stability and productivity of agricultural landscapes over the long term.

Genetic interventions, which target plant traits that affect soil structure and stability, provide creative ways to support soil conservation efforts.^6,12,15^ Changing the characteristics of roots is one way to improve soil porosity and aggregation.^51^ Deeper and more expansive root systems enable plants to reach deeper soil layers, which reduces compaction-induced soil erosion and allows for improved soil aeration and water infiltration.^52^ Furthermore, genetic alterations targeted at augmenting organic compound exudation from roots can foster the formation of soil aggregates, thereby enhancing soil stability and structure.^53–55^ These substances function as binding agents, making it easier for stable soil aggregates to form, which prevent erosion and preserve soil integrity (Fig. 1a, b). Furthermore, by encouraging beneficial microbial communities that support soil aggregation and nutrient cycling, genetic interventions focusing on plant-microbe interactions can improve soil structure.^56,57^ Genetic approaches improve soil fertility and stability by optimizing plant-microbe symbiosis, which lowers the risk of erosion and supports sustainable soil management techniques.^58^

Furthermore, targeted changes are made to plants’ genetic makeup to improve the efficiency with which they absorb vital nutrients from the soil.^6,59^ For example, one possible strategy is to modify the root hair morphology to enhance its surface area to absorb nutrients.^60,61^ A different tactic is to increase the expression of genes in plant roots that code for nutrient transporters.^6,62^ These transporters help certain nutrients, like K, P, and N, enter root cells from the soil solution. Plants can improve their capacity to take up nutrients from the soil by raising the expression levels or efficiency of these transporters (Figure 1(a,b)). Moreover, genetic modifications have the potential to enhance the mutualistic associations between plants and advantageous soil microorganisms, including rhizobia and mycorrhizal fungi.^58,63–65^ Via processes like improved N fixation and mineral solubilization, these microbes form associations with plant roots that increase nutrient uptake. GMs have the potential to improve the efficiency of symbiotic interactions, thereby facilitating improved nutrient acquisition by plants.^66^ GM strategies may facilitate the growth of bigger, more effective vascular networks in roots, which will speed up and improve the flow of nutrients all over the plant.^47,67,68^ In general, genetic modifications targeted at strengthening root systems present viable approaches to raising plant nutrient transport efficiency, which raises crop yield and nutritional quality. Therefore, by enhancing root traits, encouraging soil aggregation, and optimizing plant-microbe interactions, these genetic approaches hold promise for enhancing soil structure and conservation, ultimately supporting the long-term sustainability of agricultural ecosystems.

## 3. Case Studies and Examples of Using Genetic Intervention to Improve Soil Conservation and Nutrient Uptake

In many ecosystem processes, roots serve as a vital interface between a plant and the soil. Notably, a number of ecosystem services that cover crops offer manifest immediate benefits, including a marked decrease in soil erosion, N leaching, and weed suppression.^69^ The creation of cover crops with improved root systems represents one example of a successful use of GM techniques for soil conservation. Some species of cover crops, like grasses and legumes, have been GeMo by researchers to develop larger and deeper root systems (Figure 1(a,b)).^70,71^ For instance, through genetic engineering techniques and mutation breeding, the recently discovered *DEEPER ROOTING 1* (*DRO1*), which affects plant root systems and controls grain yield under drought stress conditions in rice, has demonstrated various benefits in improving resistance to abiotic stresses like salt and drought for other plant species, including Arabidopsis and *Prunus domestica* (*plum*)^72–74^ (Table 1). Overexpressing *PpeDRO1* in *P. domestica* (*plum*) has been demonstrated to result in deeper-rooting phenotypes, suggesting a potential use for *DRO1*-related genes to modify root architecture, even though more research is required to determine whether deeper rooting in *PpeDRO1* OE plums confers drought avoidance.^72^ Furthermore, Sun et al. demonstrate that while lateral root number was increased, lateral root angle, lateral branch angle, and silique angle were all reduced in Arabidopsis plants overexpressing potato *StDRO1*.^74^ Moreover, additional research has revealed that the *DRO1* homologs, like *qSOR1* (*quantitative trait locus for SOIL SURFACE ROOTING 1*), are useful targets for breeding root system architecture and may enhance rice yield in abiotic stress-prone conditions.^73^ Therefore, genetic engineering or editing of *DRO1* gene may enhances soil conservation by optimizing root architecture in cover crops. These GeMo cover crops improve soil structure and stability by penetrating deeper into the soil profile and offering efficient ground cover to prevent soil erosion. When compared to traditional cover crops, these GeMo cover crops have been demonstrated in field trials to dramatically lower soil erosion rates.^71,84^ Another instance is the genetic modification of crop plants to generate root exudates that encourage soil stability and aggregation.^76,85,86^ One of the key elements of plant root exudation are flavonoids, which have a variety of effects on the functioning of plant-associated microbes aside to their contribution to symbiosis. Yan et al., for instance, modified a rice flavone biosynthetic pathway using clustered regularly interspaced short palindromic repeats (CRISPR)-associated endonuclease Cas9 (CRISPR-Cas9) – based gene editing, producing apigenin-enriched rice plants that extruded apigenin into the rhizosphere. *CYP75B3* and *CYP75B4*, which encode flavonoid 3′-hydroxylases and mediate the transition of apigenin to luteolin in Kitaake rice plants, were knocked out using the CRISPR-Cas9 technique. Their research revealed that the *crispr* rice lines’ root extracts and exudates had significantly higher levels of apigenin than did the Kitaake control plants. They concluded that manipulating the flavone biosynthetic pathway is a workable and universal method for inducing biological N fixation in cereals through biofilm formation in soil diazotrophs^76^ (Table 1). Researchers have increased soil aggregation, decreased the risk of soil erosion, and improved water infiltration rates by engineering crops to release particular organic compounds from their roots.^87^ These investigations proved how well these GeMo crops work to improve soil conservation and lessen erosion on farmland.

**Table 1.** Showing examples of how genetic intervention improves soil conservation and nutrient uptake.

Plant	Traits/Genes	Technique used	Agriculture benefits	References
Rice (*Oryza sativa), Arabidopsis* and *Prunus domestica* (*plums*)	*DEEPER ROOTING 1 (DRO1)*	Transgenic approach and phylogenetic analysis	Modify the root architecture to prevent abiotic stress and make better use of resources.	^72,73,75^
Rice	Flavone biosynthetic pathway	CRISPR-based gene editing	Reduce the application of inorganic N fertilizers and promoting biological N fixation in cereals.	^76^
	Nitrate Transporter 1/Peptide Gene *OsNPF7.6*	Transgenic approach	Enhance the nitrate uptake rate of rice	^77^
	MYB transcription factor *OsMYB305*	Transgenic approach	Increasing rice’s absorption of nitrogen by lowering the flow of carbohydrates toward the production of cell walls.	^78^
Rice, barley (*Hordeum vulgare*, cv. Golden Promise, and Wheat (*Triticum aestivum*, cv. Gladius)	Alanine Aminotransferase	Transgenic approach	Offers thorough genetic and metabolic profiling of the reactions to *AlaAT* overexpression and identifies various elements and pathways that lead to the phenotype of nitrogen-use efficiency.	^79^
Maize (*Zea mays* L.) Maize	*zmm28* transcription factor	Transgenic approach	Increasing maize’s efficiencies in the uptake and utilization of N	^80^
	*CYTOKININ OXIDASE/DEHYDROGENASE* (*CKX*)	Transgenic approach	Provide ability to modify shoot element composition and engineer the root system of maize.	^81^
Winter wheat (*T. aestivum*) variety Kenong 199 (KN199)	ABRE-binding factor (ABF)-like leucine zipper transcription factor *TabZIP60*	RNA interference (RNAi)	Enhance wheat yield and efficiency in N use.	^82^
Winter wheat	Plastidic glutamine synthetase isoform (GS2)	Transgenic approach	Provide the significance of utilization of allele *TaGS2-2Ab* in enhancing the efficiency and yield of N usage in wheat.	^83^

The development of GeMo maize (corn) varieties with boosted root systems is also another example of how GM can be used to increase nutrient uptake.^80,88^ Maize has been genetically modified by researchers to develop more extensive and deeper root systems, which enable plants to absorb nutrients from lower soil layers.^51,81,89^ The efficiency of nutrient uptake was significantly enhanced by these GeMo maize varieties, especially for nutrients like phosphorus and N. For example, in one work, Fernandez et al. examined the effects on maize yield productivity caused by prolonged expression of the transcription factor *zmm28* (Event DP202216). DP202216 transgenic hybrids’ maize N uptake and utilization were examined in relation to wild-type (WT) controls. When compared to wild-type (WT) controls, DP202216 demonstrated improved N use efficiency due to increases in both N fertilizer recovered by the crop and N utilization efficiency. This study suggested that, regulating zmm28 expression provide maize breeding programs worldwide with a new and improved way to increase N utilization under diverse environmental circumstances.^80^ Moreover, Remireddy et al. demonstrate that transgenic maize lines expressing the Arabidopsis *CYTOKININ OXIDASE/DEHYDROGENASE* (*CKX*) gene at the root specific level showed up to 46% greater root dry weight although the shoot growth of transgenics lines were similar compared to WT control plants. Further, they discovered that the leaves of transgenic lines had higher concentrations of a number of elements, particularly those with low soil mobility (K, P, Mo, and Zn). Their research revealed that maize root system engineering and modification of shoot element composition could be achieved through root-specific expression of a *CKX* gene^81^ (Table 1). Another instance is the GeMo rice which enhances the expression of genes that code for root cell nutrient transporters.^18,77,78,90,91^ The GeMo rice plants are capable of absorbing and transferring nutrients more effectively, increasing yields and improving nutritional quality. This is achieved by increasing the activity of these transporters. Zhang and colleagues, for instance, have demonstrated that rice can absorb more nitrate when the nitrate transporter 1/peptide gene OsNPF7.6 is overexpressed. Their field tests also revealed that overexpressing *OsNPF7.6* increased the number of tillers overall per plant and the weight of grain per panicle, which improved rice’s agronomic nitrogen use efficiency (NUE) and grain yield. Moreover, Wang et al. showed that tiller number, shoot dry weight, and total N concentration were all markedly elevated by *OsMYB305* overexpression. They also discovered that, despite the up-regulation of N transporters (*OsNAR2.1, OsNRT2.1, OsNiR2, OsNRT2.2*) expression in the roots of *OsMYB305*-overexpression rice lines and a substantial rise in 15NO_3_^−^ influx, cellulose biosynthesis was suppressed in low-nitrogen environments. According to their findings, *OsMYB305* overexpression enhanced rice growth in low-nitrogen environments by promoting nitrate uptake through modifications in the metabolism of carbohydrates. The study suggests a novel approach to enhance rice’s uptake of nitrogen by decreasing the flux of carbohydrates toward the synthesis of cell walls.^78^ Furthermore, GeMo rice lines overexpressing the barley alanine aminotransferase (*HvAlaAT*) were used in a study by Tiong et al. to facilitate the characterization of pathways leading to more effective nitrogen use. The above-ground biomass of *OsAnt1:HvAlaAT* lines has risen under the control of the stress-inducible promoter *OsAnt1*, with minimal changes to the rates of nitrate and ammonium uptake. They also discovered that, in genetically engineered barley and wheat crops, the *OsAnt1:HvAlaAT* transgene enhanced seed production in regulated environments^79^ (Table 1). Moreover, GM has been employed to enhance the mutualistic connections between plants and advantageous soil microorganisms, like mycorrhizal fungi.^63,92,93^ Plants can be engineered to form more productive symbioses with these microbes, which will improve nutrient uptake and plant growth. These case studies collectively show how GM can enhance crop nutrient uptake, providing viable means of raising agricultural productivity and sustainability.

NUE wheat varieties are one effective example of how GM technology can be used to improve crop nutrient utilization.^6,94,95^ Scientists have discovered genes related to the uptake and metabolism of N and have incorporated them into wheat varieties. For example, Yang et al. have demonstrated recently that wheat grain yield and N use are improved by downregulating the expression of a nitrate-responsive bZIP transcription factor. In that study, it was discovered that overexpression of *TabZIP60-6D* had the contrary effects of knockdown of *TabZIP60* through RNA interference (RNAi), which increases spike number, lateral root branching, N uptake, and NADH-dependent glutamate synthase (NADH-GOGAT) activity, enhancing grain yield by over 25% under field conditions^82^ (Table 1). With lower N fertilizer inputs, these GeMo wheat varieties can yield large amounts of wheat due to their enhanced N utilization efficiency (Table 1). The GM of maize to increase its efficiency in utilizing phosphorus (P) is another example.^38,96,97^ Researchers have created maize varieties that require less P fertilizer while maintaining high yields by engineering the crop to produce enzymes that improve P uptake and utilization.^98,99^ Empirical research has demonstrated that in soils deficient in P, these GeMo varieties of maize demonstrate enhanced growth and productivity.^98,99^ Moreover, GM has been applied to rice and soybeans to boost the metabolic pathways that govern nutrient assimilation and utilization, improving crop performance and nutrient use efficiency.^67,100–102^ These examples of success show how GM can improve crop nutrient utilization, providing viable answers for environmentally friendly farming practices.

## 4. Difficulties and Aspects When Applying GM Techniques to Enhance Soil Conservation, Nutrient Uptake and Utilization

Many ethical and environmental issues are brought up by the application of GM techniques to enhance soil conservation, nutrient uptake, and utilization. First, the release of genetically modified organisms (GMOs) into the environment may have unforeseen consequences that raise concerns.^103,104^ In natural ecosystems, cross-pollination with wild or non-GMO crops may cause gene flow and inadvertent genetic changes that could upset the ecosystem^105,106^ (Table 2). Furthermore, the ownership and control of GMO seeds and crops raises ethical questions that are referred to as “biopiracy.”^107,108^ Biotechnology companies’ patenting of GMOs raises concerns about corporate monopolies in the agricultural sector and access to agricultural resources.^109,113^ Moreover, worries exist regarding genetic modification’s long-term effects on the environment, such as the possibility of inadvertent damage to non-target species, soil microbial communities, and ecosystem biodiversity.^114–116^ Overall, even though GM technology shows promise in solving certain agricultural problems, its application must be carefully considered and its ethical and environmental implications addressed to achieve fair and sustainable results.

**Table 2.** Challenges arise due to the use of genetic modification techniques for improving soil conservation, nutrient uptake, and utilization.

Challenge	Issue	Possible solutions	References
Environmental concern	Unforeseen consequences	Ensure long term safety assess of Confined Field Trials (CFTs) and proper labeling of GeMo products.	^103,104^
	cross-pollination with wild or non-GMO crops		^105,106^
Socio-economic concern	Ownership and control of GMO seeds		^107,108^
	Exploitation to the local farmers		^109^
Regulatory framework	Variation of regulatory framework from different nations		^110,111^
Public perception and opinions	Worries for long-term impacts of utilizing GM techniques		^112^

The regulatory framework controlling the application of GM techniques to enhance soil conservation, nutrient uptake, and utilization differs among nations and areas^110,117,118^ (Table 2). These approaches are covered by current legal frameworks in many jurisdictions that control the production, marketing, and distribution of genetically modified organisms.^110,119,120^ Regulators usually mandate that GeMo crop developers do thorough risk assessments to assess possible effects on the environment, human health, and socioeconomic status^6,121,122^ (Table 2). Field trials are frequently used to evaluate variables like gene stability, unintended consequences, and possible toxicity or allergenicity. Furthermore, developers might need to show that, in comparison to non-modified counterparts, their GeMo crops significantly improve soil conservation, nutrient uptake, or utilization.^6,12,13^ Moreover, GeMo products must be labeled legally in many nations to inform consumers about their inclusion in food and agricultural products.^123,124^ Generally, the regulatory environment surrounding genetically modified agriculture is intricate and dynamic, reflecting both the necessity to balance innovation with safety, environmental sustainability, and consumer transparency, as well as the continuous advancements in biotechnology.

There is continuous debate and research regarding the sustainability and long-term impacts of utilizing GM techniques to enhance nutrient uptake, conservation, and utilization of soil.^112^ Although GM technology has an opportunity to tackle agricultural challenges, its long-term effects on ecosystem health and environmental sustainability must be carefully considered to prevent the possible release of gene to unintended species.^114–116^ Furthermore, the sustainability of GeMo crops is dependent on components like their capacity to sustain productivity in the face of fluctuating environmental conditions, their influence on soil health and biodiversity, and their capacity to withstand new pests and diseases.^17,28–30^ Furthermore, broader agricultural practices and socioeconomic factors – such as market dynamics, regulatory frameworks, and resource accessibility – have a significant impact on the sustainability of GM techniques. Long-term sustainability necessitates a comprehensive strategy that takes into account the social, economic, and environmental ramifications of GMOs in addition to their technical aspects (Table 2). The sustainable application of GM techniques depends on ongoing research, observation, and adaptive management.

## 5. Future Directions

The use of emerging GM technologies has the potential to completely transform agricultural practices related to nutrient uptake, conservation, and utilization. Technological developments in gene editing provide accurate and effective ways to improve specific traits in crops, such as CRISPR-Cas9.^125,126^ Furthermore, omics technologies, such as transcriptomics, metabolomics, and genomics, allow for a thorough examination of plant characteristics associated with nutrient management and soil health.^127–129^ Moreover, gene function prediction and the creation of customized genetic modifications for particular agronomic traits are made easier by the combination of bioinformatics and computational modeling.^128,130,131^ Future GM technologies will be used to create highly productive and sustainable crop varieties that maximize nutrient uptake, conserve soil, and raise total agricultural yield.

Breeding crop varieties with improved root systems for soil stabilization and erosion control should be among the future possible uses of GM techniques for enhancing soil conservation, nutrient uptake, and utilization.^70,132,133^ Furthermore, genetic modifications can mitigate nutrient runoff and increase crop nutrient uptake efficiency, thereby decreasing reliance on chemical fertilizers.^6,134^ Moreover, genetic modifications will improve the beneficial soil microbes and plant symbiotic relationships, which will support soil fertility and nutrient cycling.^58,63–65^ Furthermore, precise modifications to metabolic pathways will be made possible by advances in gene editing technologies, which will increase crops’ efficiency in utilizing nutrients. In general, the use of GM technologies to solve agricultural problems and advance sustainable soil management techniques appears to have great potential.

The genetic underpinnings of root traits essential to soil conservation, nutrient uptake, and utilization should be the focus of future GM research priorities. While investigating interactions between GeMo crops and soil microbiota for enhanced nutrient cycling, more novel genetic tools need to be developed for precise alteration of plant traits. A thorough evaluation is necessary for the long-term environmental and socioeconomic effects, in addition to methods for combining GM technologies with sustainable farming practices.^114–116^ It is imperative to address ethical and regulatory issues, which calls for stakeholder engagement to guarantee a fair discussion about the benefits and drawbacks of GM technologies in agriculture.

## 6. Conclusion

This review demonstrated the current developments in GM techniques in tackling the urgent problems of soil preservation, nutrient uptake, and utilization in agriculture. By implementing creative genetic interventions, like techniques for decreasing erosion and improving soil structure, we have made significant strides toward preserving soil quality and addressing erosion concerns. Similarly, genetic improvements focusing on mechanisms of nutrient uptake, such as root system optimization and nutrient transport efficiency enhancement, have great potential to transform plant nutrition and boost agricultural productivity. Furthermore, genetic engineering offers promising prospects for sustainable farming practices through optimizing metabolic pathways and reducing nutrient waste through alterations in gene expression. But it’s important to recognize the long-term sustainability implications, regulatory challenges, and ethical issues related to these technologies. Going forward, the responsible and moral application of genetically modified agriculture will depend on multidisciplinary cooperation and strong regulatory frameworks. Smart crops with high nutrient absorption and utilization efficiency should be developed using future gene editing techniques, such as CRISPR-Cas 9. Effectively utilizing these developments will enable us to meet the expanding needs of a rapidly expanding global population while promoting more robust and environmentally sustainable food production systems. Fundamentally, the continuous progress in GM techniques presents a ray of hope for tackling significant agricultural issues and bringing in a new era of environmentally friendly food production.

## Data Availability

No data was generated in this study.
